# Prediction of myelosuppression risk in postoperative cervical cancer patients undergoing concurrent chemoradiotherapy using machine learning models

**DOI:** 10.3389/fonc.2026.1800585

**Published:** 2026-05-13

**Authors:** Qingkai Wang, Yaling Li, Liran Shen, Yunbiao Zhang, Qianjin Shi, Kang Shen, Hao Qiu

**Affiliations:** 1 Department of Laboratory Medicine, Shanxian Central Hospital, Heze, China; 2 Department of Clinical Medicine, School of Medicine, Nantong University, Nantong, China; 3 Department of Radiation Oncology, Siyang Hospital, Suqian, China

**Keywords:** bone marrow suppression, cervical cancer, concurrent chemoradiotherapy, dose–volume histogram, machine learning

## Abstract

**Objective:**

By integrating clinical features and pelvic bone marrow dose–volume parameters, this study developed and compared multiple machine learning models to predict the risk of bone marrow suppression (BMS) in postoperative cervical cancer patients receiving concurrent chemoradiotherapy (CCRT).

**Methods:**

A total of 415 cervical cancer patients who received postoperative CCRT at Siyang Hospital and Shanxian Central Hospital between March 2022 and April 2025 were retrospectively enrolled. The primary outcome was BMS. Candidate predictors included baseline clinical characteristics, laboratory indices, and pelvic bone marrow dose–volume parameters. Feature selection was performed using the Least Absolute Shrinkage and Selection Operator (LASSO). Logistic regression (LR), random forest (RF), Extreme Gradient Boosting (XGBoost), Light Gradient Boosting Machine (LightGBM), support vector machines (SVM), and artificial neural networks (ANN) were constructed. Model performance was evaluated in an independent test set using the area under the curve (AUC), calibration curves, and decision curve analysis, and model interpretability was assessed using SHAP.

**Results:**

Among the 415 patients, 220 (53.0%) developed BMS. Significant differences were observed between patients with and without BMS in lymph node boost irradiation, body mass index (BMI), aspartate aminotransferase (AST), serum albumin (ALB), and low-dose pelvic bone marrow irradiated volume parameters (V10, V20, and V30). LASSO retained BMI, AST, ALB, V10, V20, and lymph node boost irradiation as key predictors. Among the six models, the RF model achieved the best performance (AUC = 0.799). Calibration curves and decision curve analysis demonstrated good calibration and potential clinical net benefit of the RF model.

**Conclusion:**

Machine learning models integrating routine clinical indicators and pelvic bone marrow dose–volume parameters can effectively predict the risk of myelosuppression in postoperative cervical cancer patients receiving CCRT. The random forest model demonstrated optimal performance and may serve as a practical tool for individualised risk stratification and early intervention.

## Introduction

Cervical cancer is one of the most common malignancies among women worldwide, and standardised multimodal treatment can significantly improve patient outcomes (1). For patients with intermediate- to high-risk features after radical surgery, postoperative concurrent chemoradiotherapy (CCRT) is widely used to reduce recurrence risk and improve local control (2). However, while enhancing therapeutic efficacy, CCRT is also associated with non-negligible treatment-related toxicities, among which myelosuppression is one of the most frequent and clinically impactful complications. Myelosuppression may result in leukopenia/neutropenia, thrombocytopenia, and anaemia, increasing the risks of infection and bleeding, and potentially leading to chemotherapy dose reduction, treatment delay, or radiotherapy interruption, thereby compromising treatment intensity and tumour control outcomes (3). Previous studies have suggested that pelvic bone marrow is particularly susceptible to acute hematologic toxicity during pelvic radiotherapy combined with concurrent chemotherapy, and that the severity of toxicity is associated with bone marrow dose–volume exposure (4). Therefore, early risk assessment and stratified management of myelosuppression in patients undergoing postoperative CCRT are of substantial clinical importance.

At the level of bone marrow–sparing interventions, pelvic bone marrow–sparing intensity-modulated radiotherapy (PBMS-IMRT) has been demonstrated in prospective randomised controlled trials to reduce the incidence of ≥grade 2 or higher hematologic toxicity in cervical cancer patients receiving concurrent chemoradiotherapy, suggesting that “modifiable bone marrow dose exposure” may represent a key target for mitigating myelosuppression risk (5). Meanwhile, the international multicenter phase II INTERTECC-2 study systematically evaluated the clinical feasibility and toxicity of bone marrow–sparing IMRT in the setting of concurrent cisplatin-based radiotherapy, providing an essential clinical research foundation for quantitative prediction of hematologic toxicity and individualised radiotherapy optimisation (6). However, in real-world practice, the occurrence of myelosuppression still shows marked interindividual variability, suggesting that it may be jointly driven by patient baseline status, tumour- and treatment-related factors, and complex nonlinear relationships and interactions among variables.

Currently, risk assessment of CCRT-related myelosuppression primarily relies on clinical experience, threshold-based evaluation of a limited set of indicators, or prediction models developed with traditional statistical methods. Although these approaches can identify some high-risk patients to some extent, the mechanisms underlying myelosuppression involve the joint effects of multiple factors with complex relationships; therefore, traditional models based on linear assumptions may fail to adequately capture the actual risk structure, thereby limiting predictive accuracy, stability, and generalizability. Previous work by Chen et al. demonstrated that the associations between irradiated volumes of different bone marrow subregions at varying dose levels and hematologic toxicity are not entirely consistent, further complicating precise risk modelling (7). Therefore, there is a clear need to develop models capable of handling nonlinearity and high-dimensional interactions to achieve more reliable individualised risk prediction.

In recent years, machine learning (ML) approaches have been increasingly applied to predict treatment-related toxicity in oncology due to their advantages in handling high-dimensional features, recognizing nonlinear patterns, and capturing complex interactions among variables (8–10). Compared with traditional regression models, ML algorithms can learn more complex mappings from real-world data without relying on strict linear assumptions, thereby improving predictive performance (11–13). Moreover, advances in interpretable machine learning enable these models to provide clinically meaningful risk explanations while maintaining robust performance. Yue et al. attempted to integrate dosimetric and radiomic features to develop predictive models for acute hematologic toxicity associated with chemoradiotherapy in cervical cancer, suggesting the feasibility and potential clinical utility of ML-based, individualised risk assessment (14).

Based on this background, the present study included 415 postoperative cervical cancer patients receiving concurrent chemoradiotherapy from Siyang Hospital and Shanxian Central Hospital and integrated multidimensional features routinely available in clinical practice, encompassing patient general condition and nutritional status, tumour pathology and treatment-related information, baseline biochemical and inflammatory indicators, and radiotherapy dose–volume parameters. Six machine learning models were developed and compared to predict the risk of myelosuppression. The model with the best overall performance was selected as a basis for individualised risk assessment and early intervention in clinical practice.

## Methods

### Study population

We retrospectively collected data from cervical cancer patients who attended the oncology centres of Siyang Hospital (Suqian, Jiangsu Province) and Shanxian Central Hospital (Heze, Shandong Province) and received postoperative CCRT between March 2022 and April 2025. Data were obtained from the electronic medical records, laboratory information, and radiotherapy planning systems of both institutions and de-identified before being pooled for model development and evaluation. The study was approved by the institutional ethics committee (approval No. LL2025-045), and informed consent was waived due to the retrospective design.

**Figure 1:**
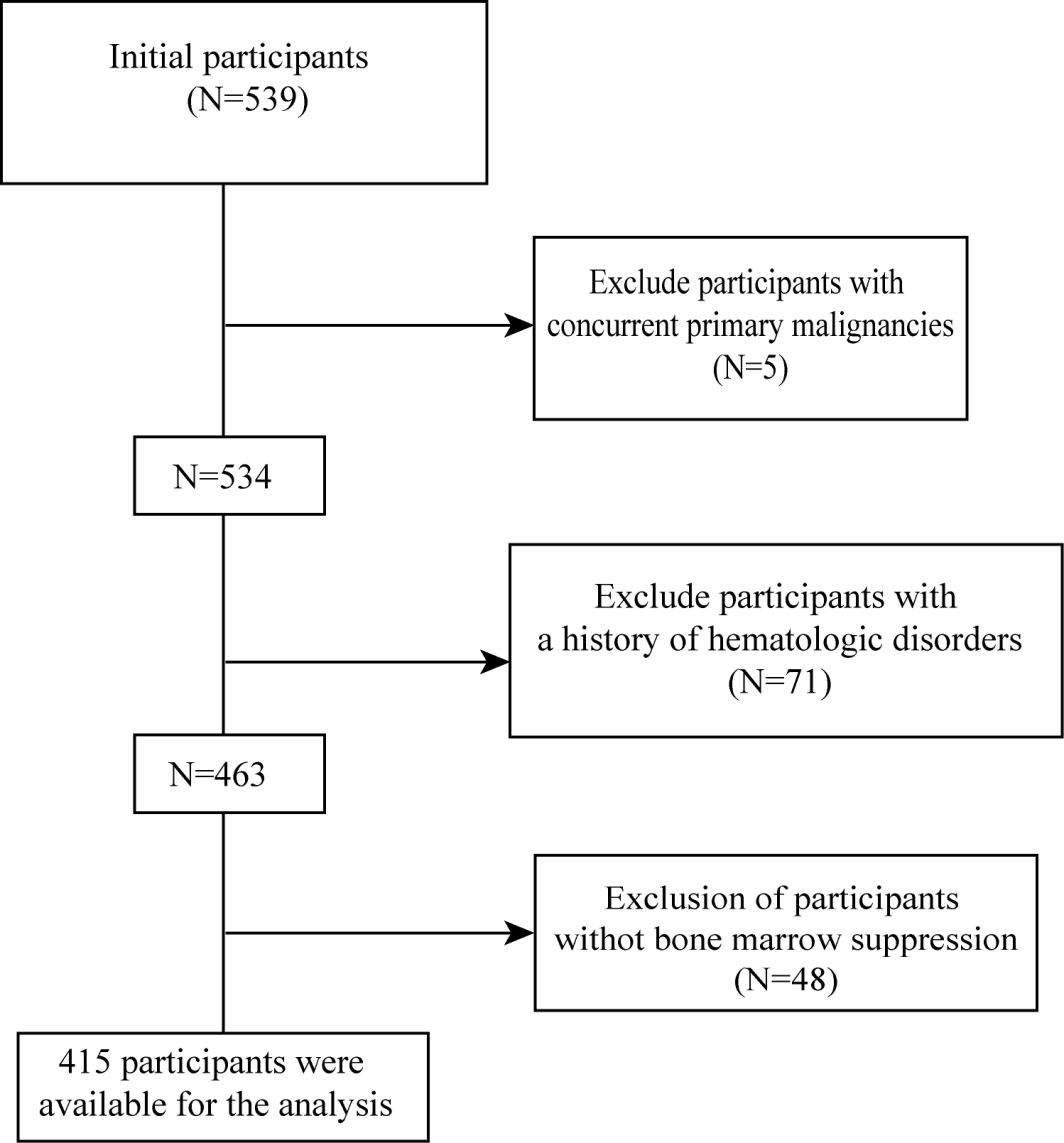
Flowchart of participant selection.

A total of 539 patients who met the postoperative treatment pathway criteria and received radiotherapy were initially screened. Inclusion criteria were as follows: (1) pathologically confirmed cervical cancer; (2) receipt of concurrent chemoradiotherapy after radical surgery; (3) availability of baseline complete blood count results before initiation of CCRT and at least one follow-up complete blood count during the observation window for outcome assessment; and (4) complete clinical data allowing extraction of variables required for model construction. Exclusion criteria included: (1) Concurrent other primary malignancies (N = 5); (2) Previously documented hematologic disorders or severe bone marrow dysfunction (N = 71); (3) Missing bone marrow suppression data (N = 48) (**Figure 1**).

### Outcome definition

The primary outcome was the occurrence of bone marrow suppression (BMS) within the observation window. BMS was defined according to the National Cancer Institute Common Terminology Criteria for Adverse Events (NCI-CTCAE) version 5.0 hematologic toxicity grading, based on white blood cell count (WBC), haemoglobin (Hb), neutrophil count (NEUT), and platelet count (PLT).

The observation window was defined as the period from the initiation of CCRT to 14 days after its completion. Initiation of CCRT was defined as the date of the first radiotherapy fraction, and completion was defined as the date of the last radiotherapy fraction; the window was extended by 14 days to capture delayed BMS events. For each patient, the worst value of WBC, Hb, NEUT, and PLT within the window (corresponding to the lowest count and highest CTCAE grade) was extracted and mapped to the Criteria for Adverse Events (CTCAE) grading system.

BMS is assessed according to the Common Terminology CTCAE version 5.0 from the National Cancer Institute. Grade 2 hematologic toxicity is defined as: hemoglobin <10.0 and ≥8.0 g/dL, white blood cell count <3.0 and ≥2.0 ×10^9^/L, absolute neutrophil count <1.5 and ≥1.0 ×10^9^/L, or platelet count <75 and ≥50 ×10^9^/L. During the observation window, hematologic suppression is defined as any of the above hematologic parameters reaching CTCAE ≥ Grade 2.

### Predictor variables and data collection

Candidate predictor variables for model development were extracted from the electronic medical records, laboratory information systems, and radiotherapy planning systems of the two institutions. To avoid information leakage, all clinical and laboratory variables were restricted to baseline data collected before initiation of CCRT; radiotherapy-related dosimetric parameters were obtained from the treatment planning system and reflected the planned dose distribution at the time of plan generation. Candidate variables mainly included demographic and anthropometric information (age, BMI), tumor-related characteristics (tumor size, pathological type), nutritional and inflammatory indicators [nutritional status, albumin (ALB), C-reactive protein (CRP)], hepatic and renal function as well as metabolic/electrolyte indices [ALT, AST, GGT, serum creatinine (Scr), uric acid (UA), potassium (K), sodium (Na), and chloride (Cl)], and radiotherapy planning and dosimetric variables (lymph node boost irradiation, target volume, and bone marrow dose–volume parameters).

To minimize cross-center information bias and ensure overall reproducibility, both participating institutions strictly adhered to standardized national radiotherapy protocols and unified organ-at-risk (OAR) contouring guidelines. Specifically, the pelvic bone marrow (PBM) was delineated on contrast-enhanced planning CTs as the low-density medullary cavities of the pelvic skeleton (encompassing the ilium, ischium, pubis, sacrococcyx, and proximal femora down to the lesser trochanter), with all historical contours retrospectively verified by a senior radiation oncologist before dosimetric extraction. Concurrently, cross-center measurement consistency for all BMS-defining laboratory parameters was ensured by using rigorously calibrated automated analyzers that underwent routine external quality assessments. Following these rigorous quality controls, the target volume was defined according to the radiotherapy planning system records and expressed in cubic centimetres (cm³). Bone marrow dosimetric parameters were then calculated based on the verified PBM structure, with V10, V20, V30, V40, and V50 representing the percentage of PBM volume receiving doses≥10, 20, 30, 40, and 50 Gy, respectively, to quantify bone marrow irradiation and evaluate its association with myelosuppression risk.

### Data preprocessing

To ensure stability and reproducibility in model training, a unified data preprocessing step was performed before model development. First, variable names and units were standardised, and continuous variables were checked for consistency within clinically plausible ranges. Extreme values that were clearly outside physiological ranges or suggestive of data entry errors were verified against original medical or laboratory records; values that could not be confirmed were treated as missing. Categorical variables (e.g., pathological type, nutritional status, and lymph node boost irradiation) were encoded as follows: unordered variables were one-hot encoded, and ordered variables were encoded using ordinal levels.

Missing data were handled using multiple imputation, with the proportion of missingness for each variable and the imputation settings reported. Variables with ≥10% missing values and unclear clinical relevance were excluded from model construction. All preprocessing steps requiring parameter estimation were performed exclusively in the training set, and the learned transformations were subsequently applied to the test set.

Given the varying sensitivity of different models to feature scales, continuous variables were scaled to improve training efficiency and convergence stability for models such as support vector machines and neural networks. Tree-based models (random forest, XGBoost, and LightGBM), which are scale-invariant, could share the same preprocessing pipeline. Ultimately, a structured feature matrix suitable for direct model input was generated, providing a consistent data foundation for subsequent feature selection, hyperparameter optimisation, and model evaluation.

### Model development and validation

Feature selection was performed using the Least Absolute Shrinkage and Selection Operator (LASSO). The regularisation parameter was determined through 10-fold cross-validation to control overfitting, yielding the final feature set for model development. The dataset was randomly split into training and test sets at a 7:3 ratio, with the training set used for model training and tuning, and the test set reserved for independent performance evaluation. Given that the incidence of BMS was 53.0% in our cohort, no substantial class imbalance was observed. Accordingly, SMOTE or other synthetic oversampling strategies were intentionally not applied, and models were trained using the original class distribution to preclude artificial noise and overfitting.

Based on the selected features, six prediction models were developed and compared: LR, RF, XGBoost, LightGBM, SVM, and ANN. Within the training set, hyperparameter optimisation was performed using a grid search with five-fold cross-validation, and the final models were trained using the parameter combinations that achieved the best cross-validation performance. To avoid data leakage, hyperparameter optimization was performed exclusively on the training set using a five-fold cross-validated grid search. All final models were then trained using the optimal hyperparameter combinations identified through cross-validation. The best hyperparameter combinations for each model are presented in **Table 1**.

**Table 1:** Optimal parameter combination for machine learning models.

Model	Optimal hyperparameters
Random Forest	n_estimators = 200; max_features = sqrt
XGBoost	colsample_bytree = 0.7; learning_rate = 0.1; max_depth = 3; n_estimators = 100; reg_alpha = 0; reg_lambda = 1.5; subsample = 0.9
LightGBM	colsample_bytree = 0.8; learning_rate = 0.1; n_estimators = 100; num_leaves = 31; subsample = 0.6
SVM	C = 0.1; degree = 2; gamma = scale; kernel = linear
ANN	activation = logistic; hidden_layer_sizes = 100

XGBoost, Extreme Gradient Boosting; LightGBM, Light Gradient Boosting Machine; SVM, Support Vector Machine; ANN, Artificial Neural Network.

### Model performance evaluation and interpretation

After feature selection and model training, the model’s performance was evaluated on an independent test set, focusing on three aspects: discrimination, calibration, and potential clinical utility. Discrimination was assessed using receiver operating characteristic (ROC) curves and the area under the curve (AUC). Calibration was evaluated using calibration curves and the Brier score to examine the agreement between predicted probabilities and observed risks. Clinical utility was assessed using decision curve analysis (DCA) to compare net benefit across different threshold probabilities. In addition, to facilitate binary classification in clinical scenarios, class labels were generated at a fixed threshold, and the trade-off between event detection and false-positive control was further characterised using the confusion matrix and derived metrics (e.g., sensitivity, specificity, and accuracy), with patients who developed bone marrow suppression defined as the event group according to the outcome criteria.

To enhance model interpretability and clinical communicability, Shapley Additive Explanations (SHAP) were applied to the final model at both global and individual levels. At the global level, SHAP importance rankings and summary plots were used to identify the features that most significantly contributed to predictions and to visualise the overall direction of risk associated with feature values. At the feature-effect level, SHAP dependence plots were used to characterise nonlinear relationships and potential interactions between key continuous variables and model outputs. At the individual level, SHAP waterfall or force plots illustrated how each feature incrementally increased or decreased predicted risk for a given case, thereby clarifying the primary factors underlying high- or low-risk predictions. Interpretation of dosimetric variables was conducted in conjunction with the definitions of pelvic bone marrow dose–volume parameters, such as V10 and V20, which represent the percentage of pelvic bone marrow volume receiving doses at or above the corresponding thresholds.

### Statistical analysis

All statistical analyses and model development were performed using R software (version 4.5.1) and Python (version 3.14.2). All statistical tests were two-sided, and P values < 0.05 were considered statistically significant. Continuous variables following a normal distribution are presented as mean ± standard deviation (SD), whereas non-normally distributed continuous variables are summarised as median and interquartile range (IQR). Categorical variables are expressed as frequencies and percentages. Between-group differences in categorical variables were assessed using the chi-square test or Fisher’s exact test, as appropriate. Continuous variables were compared using the independent-samples *t* test or the Wilcoxon rank-sum test, depending on their distributional characteristics. During feature selection and model training, bias assessment procedures were implemented to minimise the impact of differences in feature scales and distributional heterogeneity on model performance. Ultimately, six key features were selected for the machine learning model. The predictive performance of six machine learning models was subsequently evaluated and compared to identify the optimal predictive model.

## Results

### Patient characteristics

A total of 415 patients were included in this study, with a mean age of 53.82 ± 6.71 years. According to bone marrow suppression status, patients were divided into two groups: those who developed BMS (n = 220, 53.0%) and those without BMS (n = 195, 47.0%). Significant differences were observed between patients with and without BMS in lymph node boost irradiation, low- to intermediate-dose irradiated volume parameters (V10, V20, and V30), BMI, AST, and ALB (**Table 2**).

**Table 2:** Baseline characteristics.

Variables	Tota l (n=415)	No-BMS (n=195)	BMS (n=220)	P-value
Nutritional Status, n (%)				0.975
Well-nourished	253 (60.96)	119 (61.03)	134 (60.91)	
Malnutrition	162 (39.04)	76 (38.97)	86 (39.09)	
Tumor Size (cm), n (%)				0.737
<4	172 (41.45)	83 (42.56)	89 (40.45)	
≥4	243 (58.55)	112 (57.44)	131 (59.55)	
Pathological Type, n (%)				0.027
Squamous cell neoplasms	331 (79.76)	146 (74.87)	185 (84.09)	
Adenocarcinomas	84 (20.24)	49 (25.13)	35 (15.91)	
Lymph Node Boost, n (%)				0.016
No	322 (77.59)	162 (83.08)	160 (72.73)	
Yes	93 (22.41)	33 (16.92)	60 (27.27)	
V10 (%)	84.85 ± 3.53	85.58 ± 3.52	84.21 ± 3.43	<0.001
V20 (%)	66.85 ± 3.49	67.40 ± 3.69	66.37 ± 3.23	0.002
V30 (%)	56.95 [54.84, 59.31]	56.67 [54.67, 58.58]	57.55 [54.97, 59.66]	0.039
V40 (%)	40.32 [36.84, 43.23]	40.45 [37.23, 43.34]	40.02 [36.39, 42.91]	0.381
V50 (%)	4.30 [3.40, 5.26]	4.20 [3.29, 5.27]	4.32 [3.50, 5.24]	0.778
Age (years)	53.82 ± 6.71	53.72 ± 6.41	53.90 ± 6.97	0.784
Vloum (cm^3^)	1251.00 [1141.50, 1375.00]	1260.00 [1159.50, 1385.50]	1243.50 [1105.75, 1354.00]	0.185
Scr (umol/L)	53.42 ± 4.47	53.47 ± 4.66	53.38 ± 4.31	0.836
UA (umol/L)	263.05 ± 79.50	267.36 ± 79.10	259.23 ± 79.85	0.299
K (mmol/L)	4.01 ± 0.29	4.04 ± 0.30	3.99 ± 0.28	0.126
Na (mmol/L)	138.33 ± 2.08	138.33 ± 2.16	138.33 ± 2.02	0.973
Cl (mmol/L)	103.40 [101.96, 105.30]	103.33 [101.68, 105.28]	103.73 [102.23, 105.30]	0.397
BMI (kg/m^2^)	25.00 ± 3.25	26.18 ± 3.03	23.96 ± 3.09	<0.001
ALT (U/L)	24.77 [17.14, 33.31]	24.28 [17.03, 32.34]	25.86 [17.15, 33.80]	0.848
AST (U/L)	27.07 [21.55, 33.52]	25.09 [20.51, 31.12]	29.91 [23.91, 36.05]	<0.001
ALB (g/L)	40.17 [37.72, 42.92]	40.47 [38.21, 43.08]	39.52 [37.31, 42.67]	0.042
GGT (U/L)	41.94 [22.49, 99.79]	42.00 [22.68, 95.76]	40.88 [22.40, 101.00]	0.779
CRP (mg/L)	0.73 [0.28, 1.83]	0.71 [0.30, 1.69]	0.78 [0.27, 1.87]	0.821

BMS, Bone marrow suppression; UA, uric acid; CRP, C-reactive protein; GGT, Gamma-glutamyl transferase; BMI, body mass index; ALB, albumin; ALT, alanine aminotransferase; AST, aspartate aminotransferase; Scr, serum creatinine.

### Development of models

**Figure 2:**
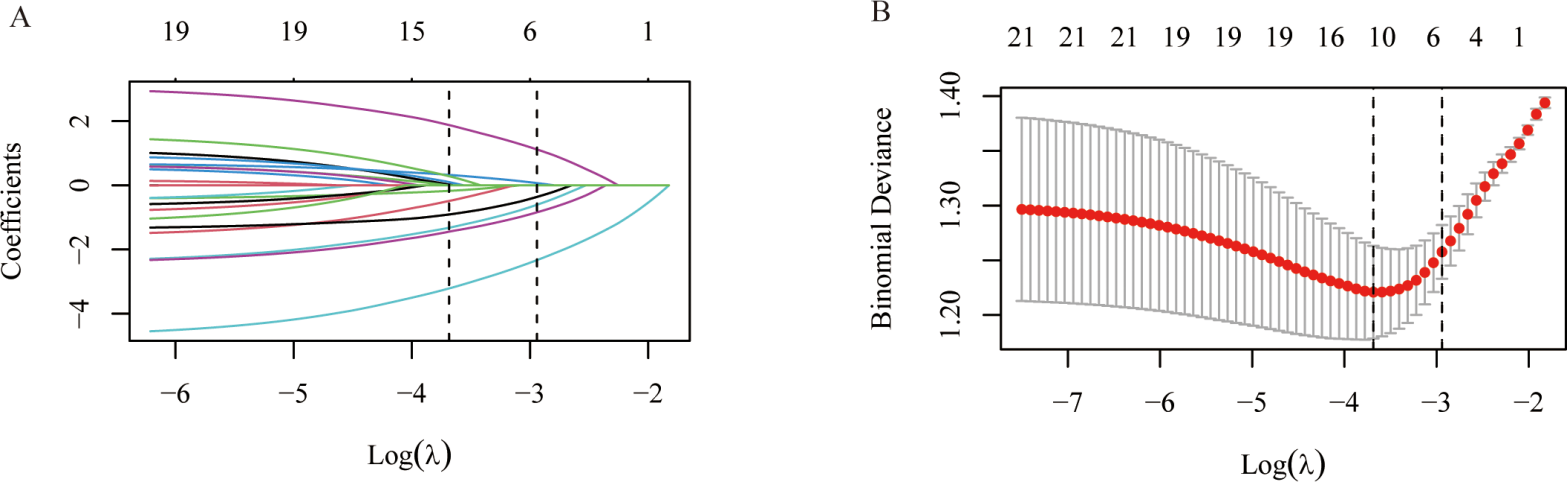
Feature selection. **(A)** The coefficient profiles of the nine features obtained through the LASSO. **(B)** The tuning parameters for the LASSO model are selected via 10-fold cross-validation. LASSO, least absolute shrinkage and selection operator.

**Figure 3:**
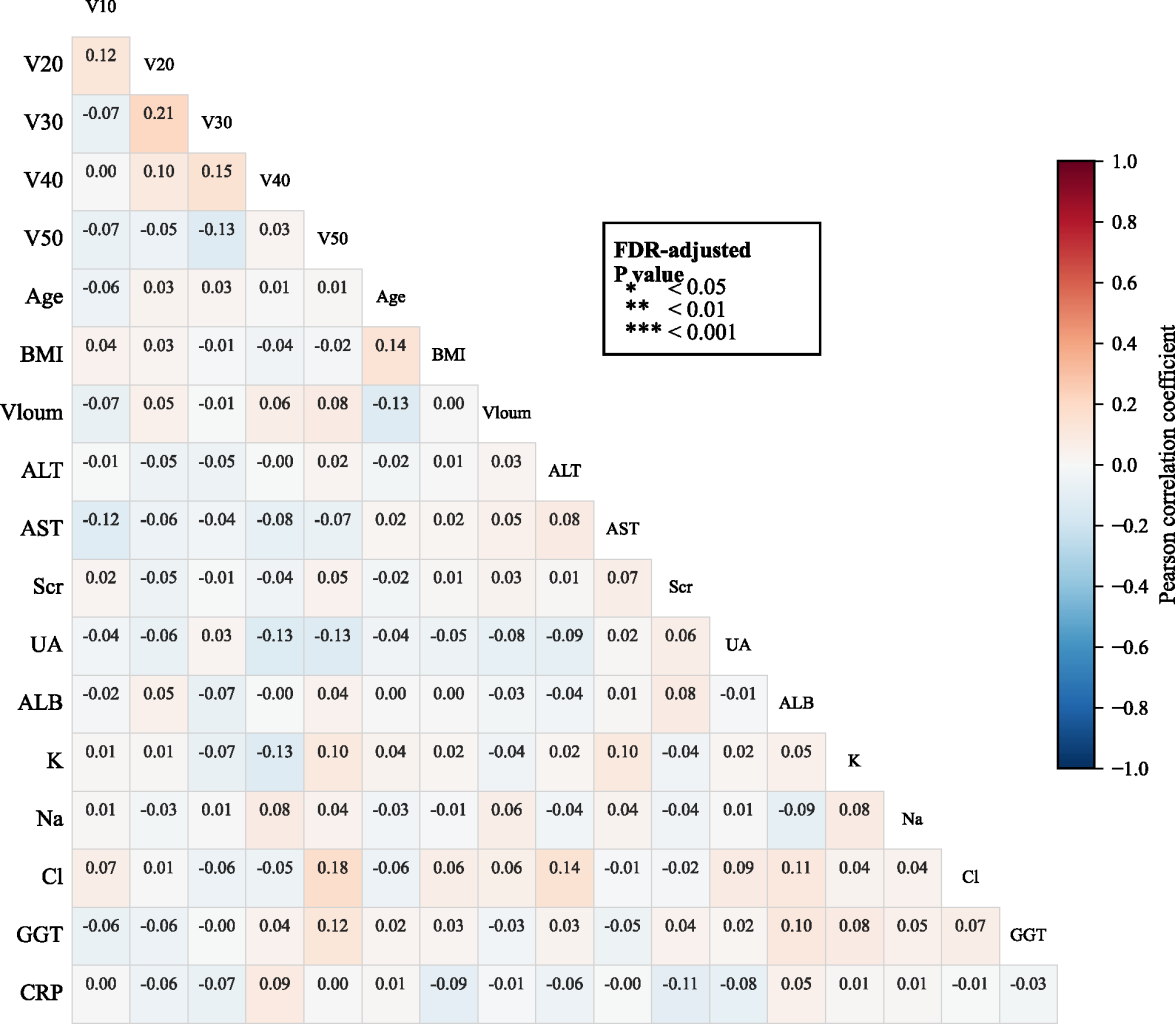
Correlation heatmap of candidate variables.

All samples were randomly divided into training and test sets at a 7:3 ratio, and variable selection was performed using LASSO regression with 10-fold cross-validation. As the regularisation parameter λ increases, the regression coefficients of most variables gradually shrink toward zero, with only a few retaining non-zero coefficients (**Figure 2A**). As log(λ) varies, the cross-validation-derived binomial error exhibits a trend of first decreasing and then increasing, reaching its minimum at the optimal λ value (λ_min) (**Figure 2B**). Based on the selected optimal λ, six key predictor variables (BMI, AST, ALB, V20, V10, lymph node boost) were ultimately screened and used to construct the subsequent machine learning model. The correlation heatmap indicates weak overall correlations among variables, with no prominent clusters of high correlations. V10 and V20 exhibit only a weak positive correlation (r = 0.12), suggesting no significant collinearity between them. Concurrently, the correlation coefficients between V10/V20 and target volume, age, BMI, and multiple laboratory indicators are all low, with no significant linear correlations detected overall (**Figure 3**).

### Evaluating ML model performance

**Figure 4:**
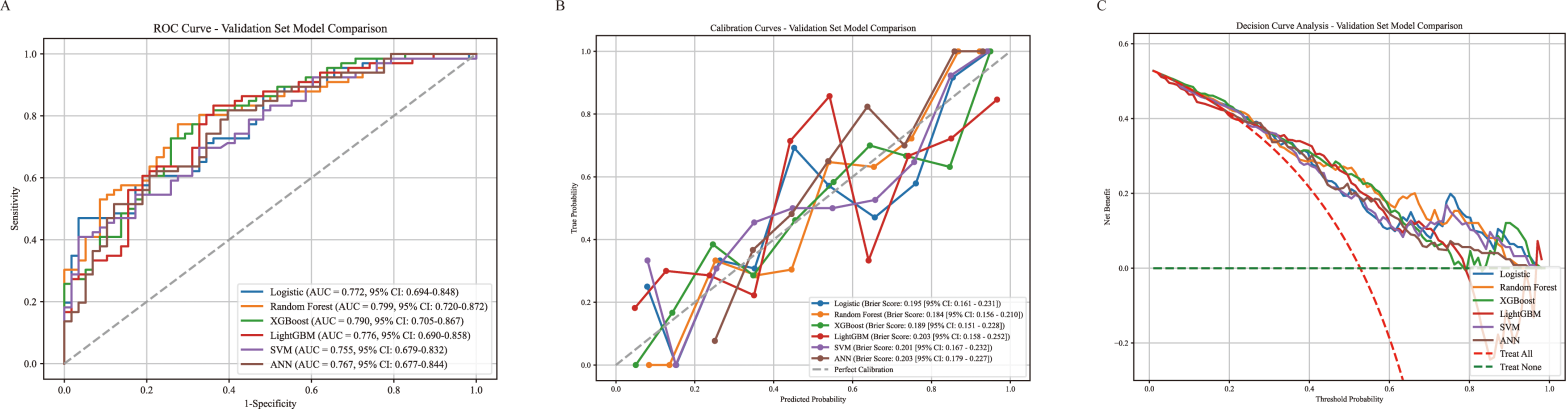
Model performance evaluation and interpretation. **(A)** ROC curves for the test dataset. **(B)** Calibration curves for the models. **(C)** DCA curves for the test dataset.

**Figure 5:**
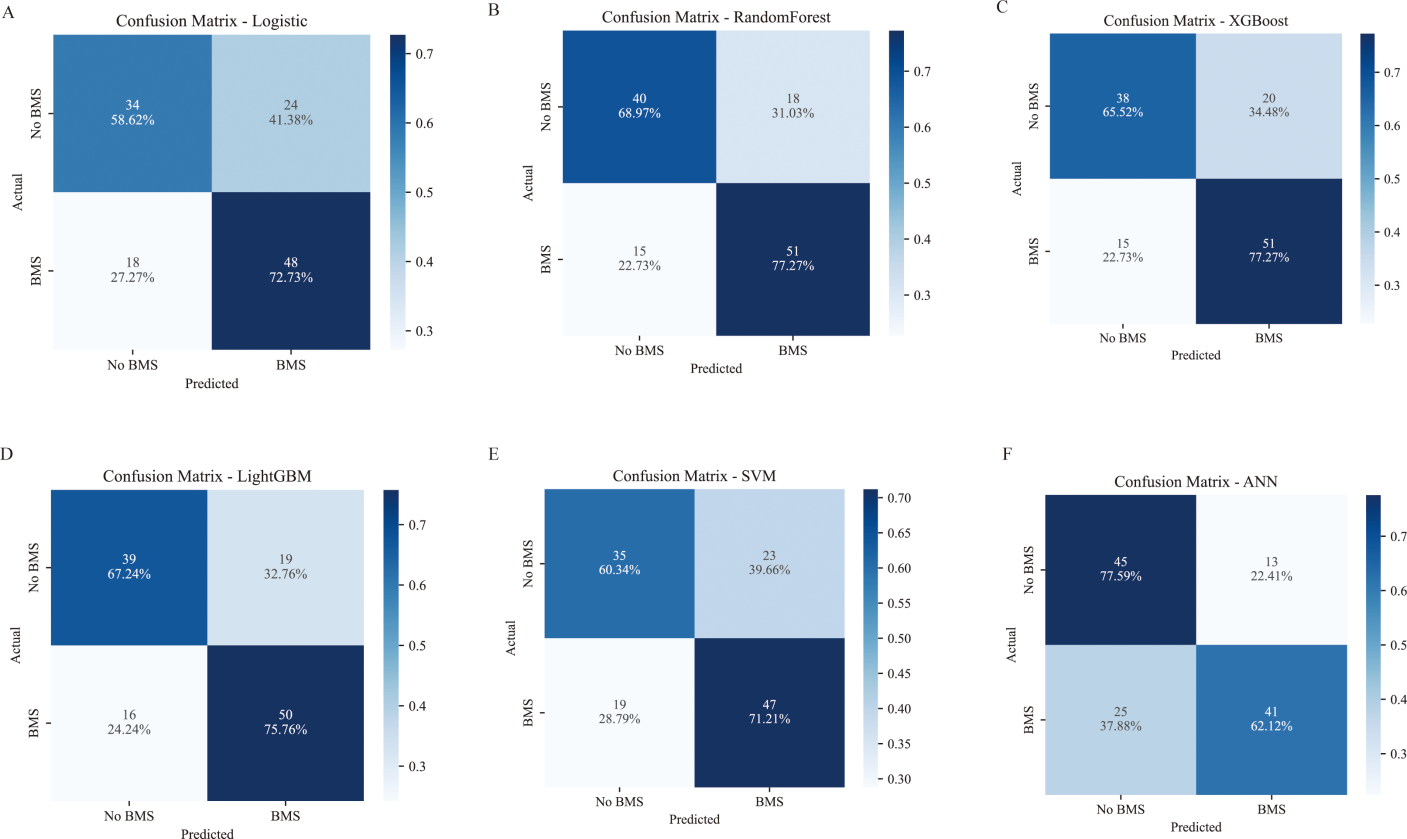
The confusion matrix for models. Figure **(A–F)** shows the confusion matrix for **(A)** LR, **(B)** RF, **(C)** XGBoost, **(D)** LightGBM, **(E)** SVM, and **(F)** ANN, respectively. BMS, Bone marrow suppression; LR, Logistic Regression, RF, Random Forest; XGBoost, Extreme Gradient Boosting; LightGBM, Light Gradient Boosting Machine; SVM, Support Vector Machine; ANN, Artificial Neural Network.

Six machine learning models were developed using the training set and evaluated in the test set. Performance metrics included the AUC, accuracy, precision, sensitivity, specificity, F1 score, positive predictive value (PPV), and negative predictive value (NPV) (**Table 3**). In terms of overall performance, the RF model achieved the highest F1 score (0.756), followed by XGBoost (0.745), LightGBM (0.741), LR (0.696), SVM (0.691), and ANN (0.683). The corresponding AUC values were 0.799 (95% CI: 0.720–0.872), 0.790 (95% CI: 0.705–0.867), 0.776 (95% CI: 0.690–0.858), 0.772 (95% CI: 0.694–0.848), 0.755 (95% CI: 0.679–0.832), and 0.767 (95% CI: 0.720–0.872), respectively (**Figure 4A**). Among all the models, the random forest model demonstrated the best overall predictive performance, achieving the highest AUC and F1 score, while also yielding the highest Kappa value (0.464) and Youden’s J index (0.462). These results indicate that the model not only had superior discriminative ability but also achieved the best balance between overall agreement and diagnostic effectiveness. In addition, while maintaining a relatively high sensitivity (0.773), the random forest model also achieved favorable specificity (0.690), positive predictive value (PPV, 0.739), and negative predictive value (NPV, 0.727), suggesting that it was effective in identifying patients at high risk of myelosuppression and also exhibited relatively stable clinical utility. Calibration curves showed good agreement between predicted and observed outcomes for all models, particularly for the RF model (**Figure 4B**). DCA further indicated that both the RF and LR models provided favourable clinical net benefit across a range of threshold probabilities (**Figure 4C**). In addition, confusion matrices for all models were presented, with the RF and XGBoost models exhibiting relatively high sensitivity (**Figure 5**).

**Table 3:** Comparative analysis of the performance outcomes across machine learning models.

Model	AUC	Precision	Sensitivity	Specificity	F1 Score	PPV	NPV	Kappa	Youden’s J
LR	0.772	0.667	0.727	0.586	0.696	0.667	0.654	0.315	0.313
RF	0.799	0.739	0.773	0.69	0.756	0.739	0.727	0.464	0.462
XGBoost	0.79	0.718	0.773	0.655	0.745	0.718	0.717	0.430	0.428
LightGBM	0.776	0.725	0.758	0.672	0.741	0.725	0.709	0.431	0.430
SVM	0.755	0.671	0.712	0.603	0.691	0.671	0.648	0.317	0.316
ANN	0.767	0.759	0.621	0.776	0.683	0.759	0.643	0.392	0.397

LR, Logistic Regression, RF, Random Forest, XGBoost, Extreme Gradient Boosting, LightGBM, Light Gradient Boosting Machine, SVM, Support Vector Machine, ANN, Artificial Neural Network, PPV, Predictive Value, NPV, Negative Predictive Value, AUC, Area Under the Curve.

### SHAP-based model visualisation

**Figure 6:**
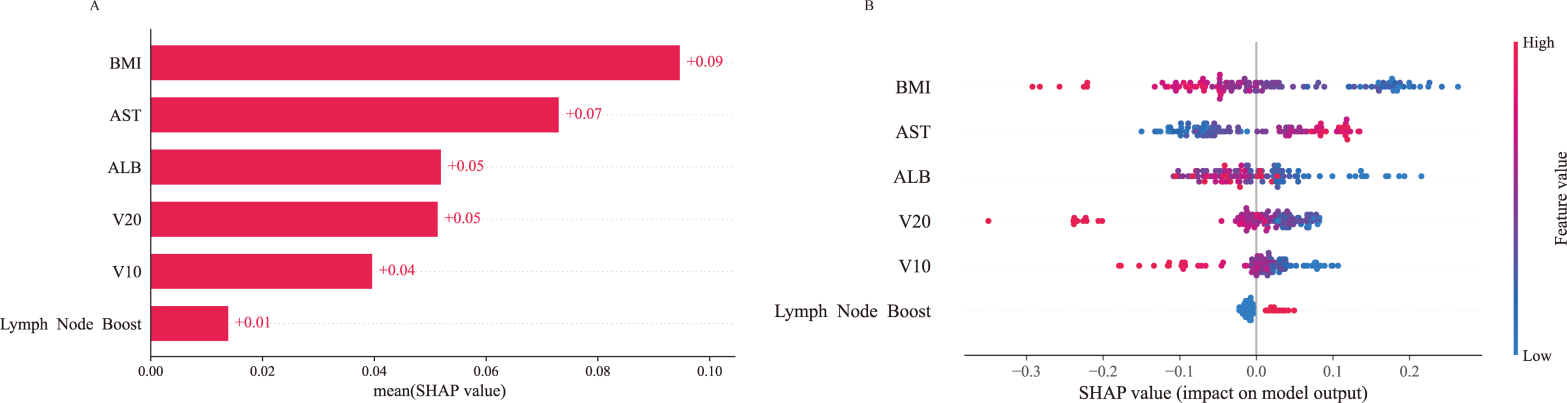
SHAP explains the results of the RF model. **(A)** SHAP values for each feature variable. **(B)** Summarises the distribution of SHAP values across all features, illustrating the magnitude and direction of each feature’s contribution to the model predictions. The colour gradient represents the corresponding feature values, with red denoting higher values and blue indicating lower values.

**Figure 7:**
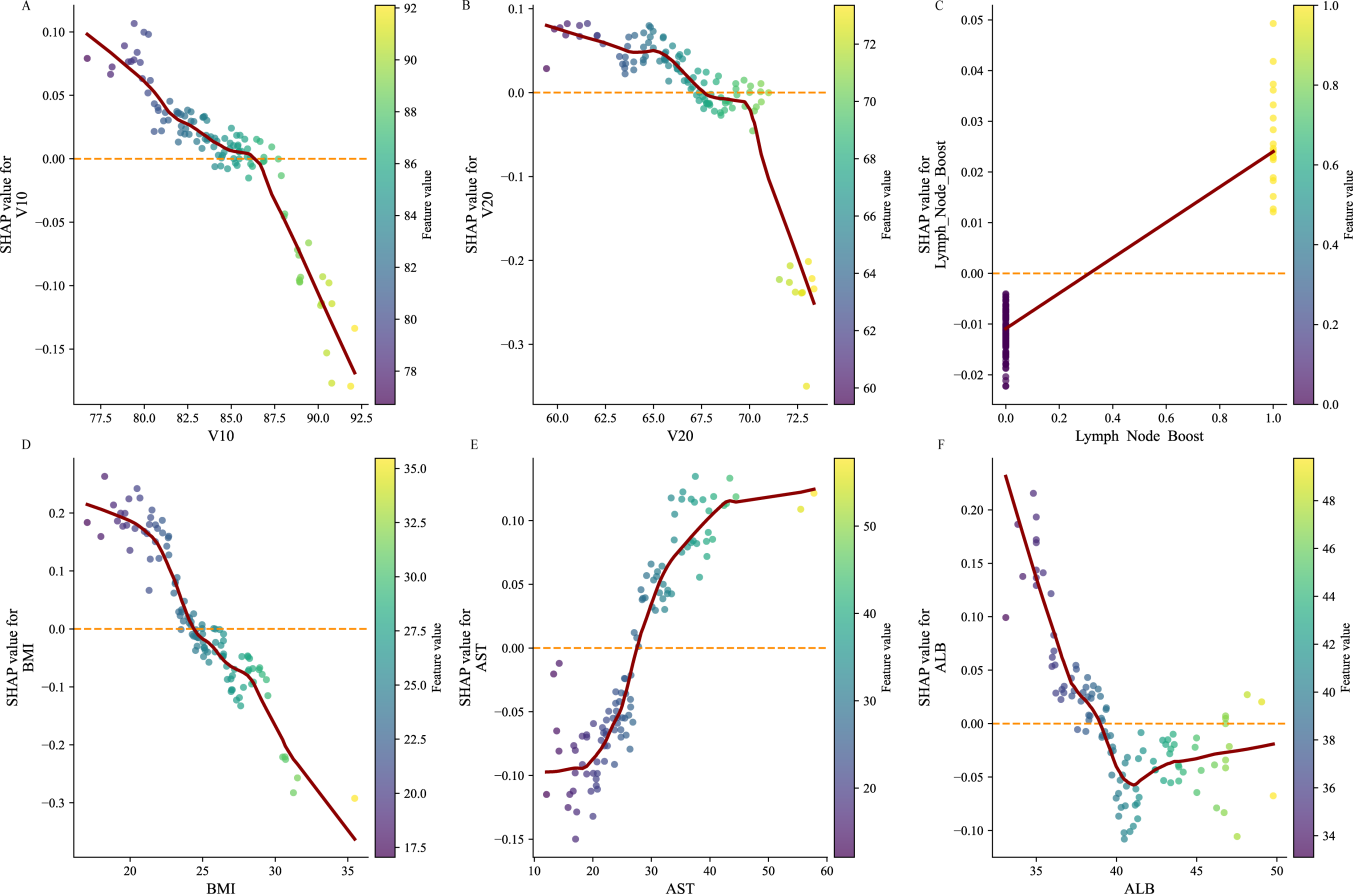
Feature dependence plots based on SHAP values. **(A)** V10; **(B)** V20; **(C)** Lymph Node Boost; **(D)** BMI; **(E)** AST; **(F)** ALB.

**Figure 8:**
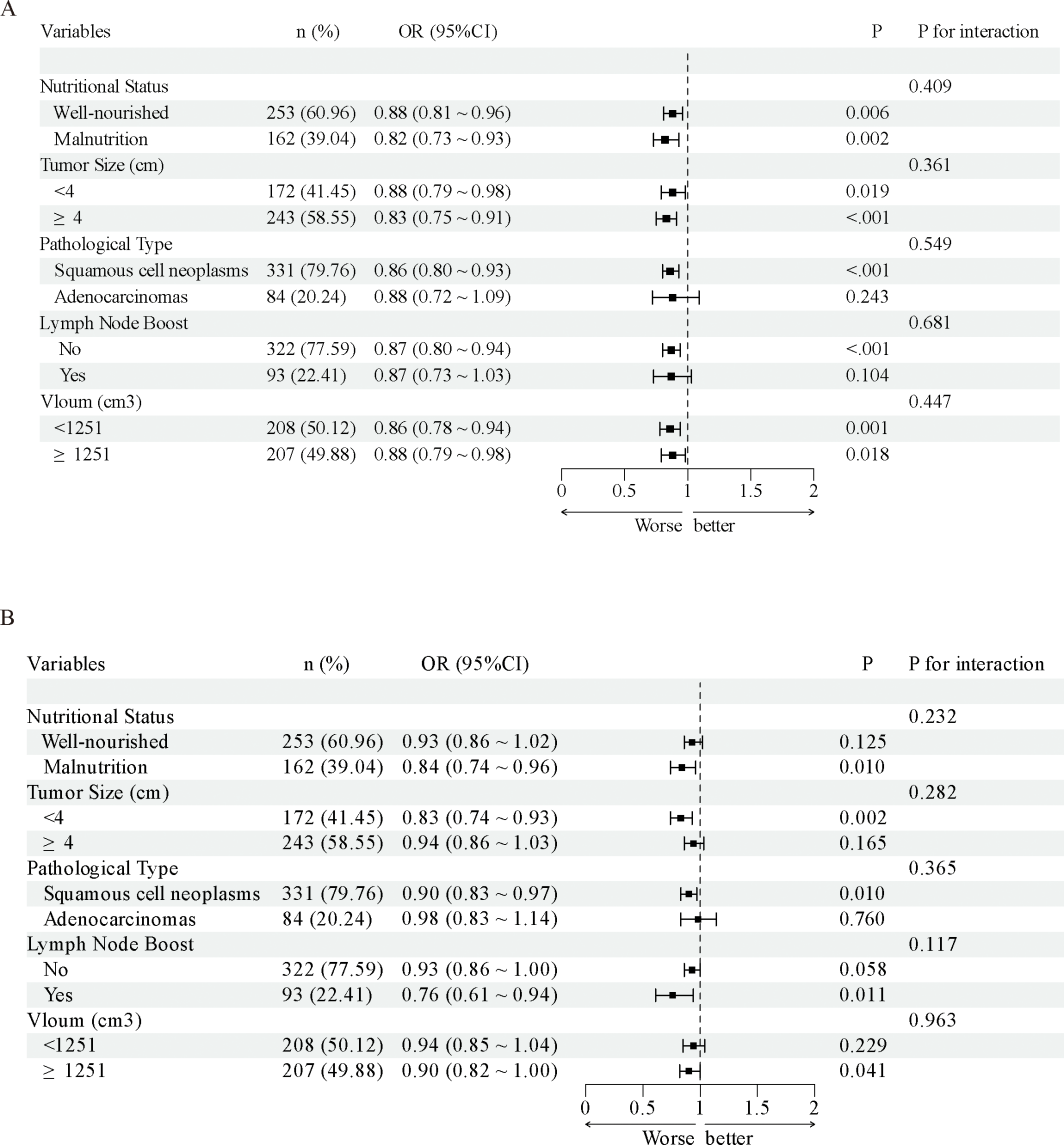
Forest plot showing the association between V10/V20 and the risk of bone marrow suppression across different subgroups. **(A)** V10 **(B)** V20.

SHAP was applied to the RF model to quantify the importance of each predictor in predicting model outcomes. The results indicated that BMI had the highest predictive importance, followed by AST, ALB, V20, V10, and lymph node boost irradiation (**Figure 6A**). Further SHAP-based risk factor analysis showed that lymph node boost irradiation had a positive effect, shifting predictions toward BMS. In contrast, higher BMI had an adverse impact, driving predictions toward the non-BMS outcome (**Figure 6B**). SHAP scatter plots of the RF model, illustrating the relationships between key feature values and their corresponding SHAP values, demonstrated marked heterogeneity in how different features influenced the predicted risk of myelosuppression (**Figure 7**). Notably, the risk of myelosuppression decreased with increasing V10 and V20. In subgroup analyses adjusted for all covariates, the inverse association between V10 and BMS remained consistent across strata defined by nutritional status, tumor size, histological type, lymph node boost, and radiotherapy volume, with ORs <1 in all strata and no significant interactions observed (all P for interaction >0.05) (**Figure 8A**). Similar results were observed for V20. Although the association did not reach statistical significance in the adenocarcinoma, no lymph node boost, and tumor size ≥4 cm subgroups, no significant interaction was identified in any subgroup (all P for interaction >0.05) (**Figure 8B**). These findings suggest that the inverse associations of V10 and V20 with BMS were generally consistent across clinically relevant subgroups and were unlikely to be fully explained by the factors examined.

**Figure 9:**
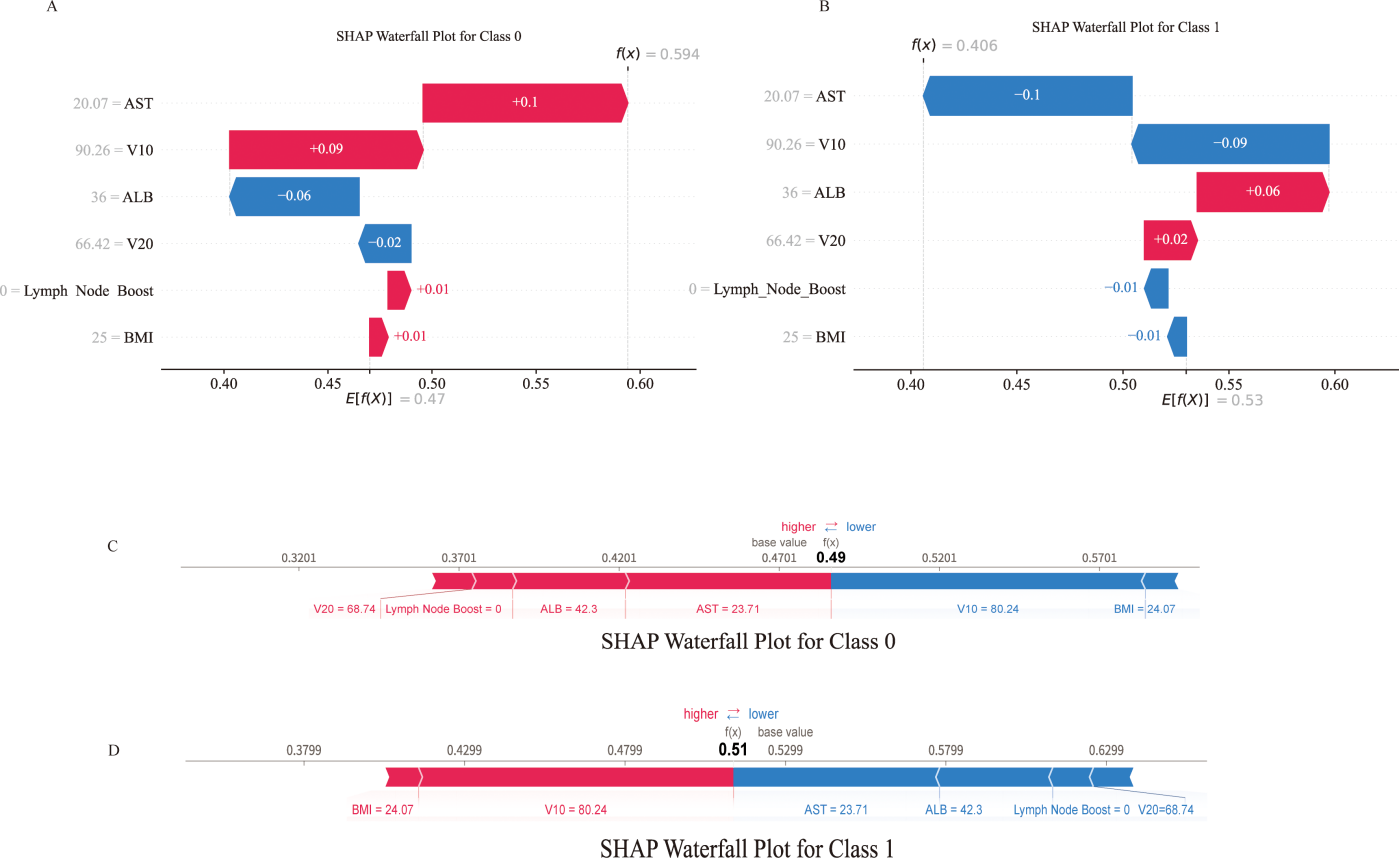
Single-sample interpretation based on the SHAP values. **(A, B)** Waterfall chart for the same patient, **(A)** Occurrence of bone marrow suppression, **(B)** No bone marrow suppression occurred. **(C, D)** Force plot for the same patient, **(C)** Occurrence of bone marrow suppression, **(D)** No bone marrow suppression occurred.

For individual-level interpretation, SHAP waterfall and force plots were generated for the same patient across different outcome categories to explain case-specific predictions. The same features exhibited distinct contribution patterns across outcomes, thereby shifting model outputs toward the presence or absence of bone marrow suppression. Features increasing the predicted risk were highlighted in red, whereas those decreasing the predicted risk were shown in blue (**Figure 9**).

## Discussion

Based on data from 415 postoperative cervical cancer patients receiving concurrent chemoradiotherapy, this study developed and compared multiple machine learning models to predict the risk of bone marrow suppression. Random forest model achieves the best overall metrics (AUC = 0.799, sensitivity = 0.773, specificity = 0.690). These findings suggest that, in real-world postoperative CCRT populations, reliable stratification of BMS risk can be achieved by integrating routinely available clinical, anthropometric, and laboratory indicators with planned dose–volume information, thereby providing a quantitative basis for early monitoring, prophylactic intervention, and management of treatment intensity. Meanwhile, the incidence of BMS in this cohort was relatively high (220/415), and several baseline variables differed significantly between the BMS and non-BMS groups, including pathological type, lymph node boost irradiation, and selected dose–volume parameters.

Previous studies have increasingly reinforced the association between pelvic bone marrow irradiation and hematologic toxicity in recent clinical research. A prospective randomised phase II trial demonstrated that pelvic bone marrow–sparing radiotherapy reduced the incidence of acute hematologic toxicity, supporting the notion that “optimisation of bone marrow irradiation” is an actionable strategy with clinical benefit (15). In parallel, growing attention has been paid to the impact of low- to intermediate-dose regions. Zhang et al. reported that pelvic bone marrow V5, V10, V20, and V30 were significantly associated with treatment-related lymphopenia, suggesting that diffuse low-dose exposure may also warrant consideration as a planning constraint (16).

Notably, in our cohort, V10 and V20 were paradoxically lower in the BMS group. We strongly emphasize that this inverse association lacks biological plausibility and does not imply that reducing low-dose irradiation paradoxically aggravates toxicity. Instead, this counterintuitive trend is driven by a combination of technical, statistical, and clinical factors. Technically, our CT-based anatomical delineation, while highly standardized, cannot exclude hematopoietically inactive yellow marrow as accurately as functional MRI does, thereby inherently introducing structural noise into the low-dose analysis (17–19). Statistically, dosimetric parameters exhibit inherent collinearity; in high-dimensional algorithms without biologically informed prior constraints (e.g., random forests), the model may exploit these complex collinear relationships to minimize mathematical error, resulting in features being assigned predictive importance in directions that are mathematically optimal but biologically contradictory (20–22). Clinically, these lower parameters likely serve as statistical proxies for specific “treatment intensities” or adaptive therapy changes—for instance, patients experiencing early hematologic decline often receive prompt concurrent chemotherapy dose reductions, temporary interruptions to radiotherapy, or aggressive supportive care. These dynamic clinical interventions actively alter the subsequent toxicity trajectory, confounding the static baseline dosimetric exposures (23, 24). Consequently, the roles of V10 and V20 here should be interpreted strictly as dataset-specific statistical signals rather than as causal biological mechanisms.

In the current landscape of predictive modeling for radiotherapy-induced hematologic toxicity, existing studies exhibit considerable heterogeneity in model performance, feature sets, and population characteristics. Previous efforts have utilized diverse algorithms—ranging from support vector machines to elastic-net logistic regression—and incorporated varying predictor sets across different gynecologic cancer cohorts, yielding predictive performances with AUCs typically spanning from 0.65 to 0.83 (25–27). Despite these advancements, a systematic consensus on an optimal, universally applicable predictive framework remains elusive. It is within this broader context that our study establishes its innovative value and relative advantage. At the level of predictive modelling, recent studies have increasingly favoured integrating dosimetric information with radiomics or imaging-derived features to enhance early prediction of hematologic toxicity. Le et al. reported a radiomics-based approach for predicting acute hematologic toxicity and highlighted radiomic features as an essential complement to conventional clinical and dosimetric information (28). In addition, Zhu et al. emphasised that, while maintaining discrimination performance, providing traceable individualised explanations using methods such as SHAP can improve the clinical usability of predictive models (29). Compared with these feature-intensive frameworks that combine imaging, dosimetry, and clinical variables, the present study emphasises a “low-threshold and implementable” strategy. The core predictors were primarily derived from routinely available BMI and biochemical indices, together with dose–volume information directly exportable from the treatment planning system, while still achieving acceptable discrimination performance. This approach may offer greater translational value in clinical settings where radiomics has not yet been fully standardised, and facilitates rapid deployment and iterative refinement within real-world workflows.

From the perspectives of variable interpretation and biological plausibility, the features retained in this study are broadly consistent with the mechanisms underlying bone marrow suppression (BMS). BMI and serum albumin reflect nutritional reserve and overall metabolic status, which are closely associated with tolerance to chemoradiotherapy and the capacity for bone marrow recovery (30, 31). Aspartate aminotransferase (AST) may, to some extent, serve as an indirect indicator of hepatic function and systemic inflammatory or metabolic stress, and its elevation may correspond to poorer treatment tolerance or a higher risk of complications (32, 33). In the baseline comparisons, BMI, AST, and ALB differed significantly between the BMS and non-BMS groups, aligning with their importance rankings in model interpretation.

Meanwhile, a higher proportion of lymph node boost irradiation was observed in the BMS group, which is also clinically plausible: boost irradiation implies a larger irradiated volume or higher local dose burden and may increase the risk of bone marrow suppression by expanding pelvic bone marrow exposure or intensifying overall treatment. Therefore, from a clinical application perspective, the proposed model may not only support risk prediction but also highlight potentially modifiable factors. For patients identified as high risk, earlier initiation of intensified hematologic monitoring and nutritional support may be considered, and more refined bone marrow–sparing strategies could be explored during radiotherapy planning to reduce treatment interruptions and the adverse impact of compromised treatment intensity.

Finally, the interpretability analysis provided critical support for the model’s clinical communication. The SHAP framework not only provided a global ranking of variable importance but also illustrated how key variables influenced the direction of risk across different value ranges, enabling clinicians to intuitively understand why a given case was classified as high or low risk. Particularly when specific dosimetric parameters exhibited inverse trends, interpretable outputs helped present the model’s learned associations transparently. They provided directions for future research, such as optimising bone marrow delineation strategies, incorporating functional bone marrow subregion dosimetric metrics, and evaluating the stability of these associations through external validation cohorts. Overall, this study provides an implementable baseline framework for individualised BMS risk prediction, but several limitations must be acknowledged. First, utilizing a 14-day composite endpoint may miss late-onset cytopenias and obscure lineage-specific predictive patterns. Second, our static baseline model fails to capture dynamic intra-treatment variables, such as temporary dose modifications or supportive care. Finally, lacking independent external validation, the model’s true generalizability remains uncertain. Therefore, large-scale, multicenter prospective validation incorporating extended follow-up and dynamic, lineage-specific modeling is strictly required before safe clinical deployment.

## Conclusion

This study developed and validated a machine learning model integrating conventional clinical indicators and pelvic bone marrow dose-volume parameters to predict the risk of bone marrow suppression in postoperative cervical cancer patients undergoing concurrent chemoradiotherapy. Among the evaluated models, RF demonstrated the best overall predictive performance and clinical utility. It provides valuable insights for clinical treatment decision-making and improving patient outcomes.

## Data Availability

The datasets used and analysed during the current study are not publicly available due to institutional and patient privacy restrictions but are available from the corresponding author on reasonable request.
